# 

**Published:** 1891-06

**Authors:** 


					JUNE, 1891.
Vol. IX. No. 32.
THE
BRISTOL
MEDICO-CHIRURGICAL
JOURNAL.
S (Siuarterlg jQurnal of tbc /ifteMcal Sciences for tbe Mest
of England and Soutb "CClales
PUBLISHED UNDER THE AUSPICES OF
THE BRISTOL MEDICO-CHIRURGICJL SOCIETY.
EDITOR :
J. GREIG SMITH, M.A., F.R.S.E.
ASSOCIATED WITH
BARCLAY J. BARON, M.B., C.M. F. R. CROSS, M.B.
J. MICHELL CLARKE, M.B., M.A. W. H. HARSANT.
ASSISTANT-EDITOR '.
L. M. GRIFFITHS.
"Scire cat ncscirc, nfsf to mc
Scirc alius scirct."
m . H
E ^ I
O ? m r
o ^ CD ?
"~T~ 20/ ^
I ' I
-<
CO
I
%
7
BRISTOL : J. W. ARROWSMITH ;
London: J. & A. Churchill, 11 New Burlington Street.
Price One Shilling and Sixpence.

				

